# Geographical location influences the composition of the gut microbiota in wild house mice (*Mus musculus domesticus*) at a fine spatial scale

**DOI:** 10.1371/journal.pone.0222501

**Published:** 2019-09-26

**Authors:** Sarah Goertz, Alexandre B. de Menezes, Richard J. Birtles, Jonathan Fenn, Ann E. Lowe, Andrew D. C. MacColl, Benoit Poulin, Stuart Young, Janette E. Bradley, Christopher H. Taylor

**Affiliations:** 1 School of Life Sciences, University of Nottingham, Nottingham, United Kingdom; 2 School of Natural Sciences, NUI Galway, Galway, Ireland; 3 School of Environment and Life Sciences, University of Salford, Manchester, United Kingdom; 4 IUCN SSC Asian Wild Cattle Specialist Group, c/o Chester Zoo, Chester, United Kingdom; University of Illinois at Urbana-Champaign, UNITED STATES

## Abstract

The composition of the mammalian gut microbiota can be influenced by a multitude of environmental variables such as diet and infections. Studies investigating the effect of these variables on gut microbiota composition often sample across multiple separate populations and habitat types. In this study we explore how variation in the gut microbiota of the house mouse (*Mus musculus domesticus*) on the Isle of May, a small island off the east coast of Scotland, is associated with environmental and biological factors. Our study focuses on the effects of environmental variables, specifically trapping location and surrounding vegetation, as well as the host variables sex, age, body weight and endoparasite infection, on the gut microbiota composition across a fine spatial scale in a freely interbreeding population. We found that differences in gut microbiota composition were significantly associated with the trapping location of the host, even across this small spatial scale. Sex of the host showed a weak association with microbiota composition. Whilst sex and location could be identified as playing an important role in the compositional variation of the gut microbiota, 75% of the variation remains unexplained. Whereas other rodent studies have found associations between gut microbiota composition and age of the host or parasite infections, the present study could not clearly establish these associations. We conclude that fine spatial scales are important when considering gut microbiota composition and investigating differences among individuals.

## Introduction

The gut microbiota interacts with a variety of fundamental host functions in most animals. It is involved in breaking down complex carbohydrates for energy [1], and immunological functions such as the activation and production of T-Regulatory cells or the induction of cytokines such as FoxP3 or IL10 [2,3]. Changes in the composition and microbial abundance of the gut microbiome occur throughout life due to factors such as changes in hormone levels [4], age [5], infection with parasites [6], diet [7] and host genetics [8,9]. Additionally, vertical transmission from mother to offspring and the transmission of bacteria from the environment, such as the vegetation, can also affect microbial composition [10–12].

One of the most influential variables on gut microbiota composition in wild house mice is their geographical provenance [13–15]. The geographical scale over which this has been studied varies markedly, having up to 100 kilometres between all of the sampling sites [13] or sampling mouse populations from different countries [14]. The caecal microbiota of house mice (*Mus musculus domesticus*) showed differences in alpha diversity across the UK using two farmland sites (in Gloucestershire and Somerset), and the London underground [13]. Geographical distance has been identified to account for 16% of gut microbiota variation in wild house mice across Western Europe [14]. It has also been suggested that microbiota variation may be limited by the dispersal abilities of the microbes themselves and the level of interbreeding and direct physical contact among the hosts [14]. Altitude has also been shown to impact the composition of wild house mouse gut microbiota due to changes in available resources for the bacteria [15]. As altitude increases, anaerobic bacteria (e.g. *Prevotella* species) increase in the rodent’s environment compared to lower altitude habitats whilst other microbes decline due to the limited oxygen available, and this influences the community which can be acquired by the host [15].

Geographical location is intimately linked with differences in ecological factors such as local vegetation, which in turn are likely to directly or indirectly affect the diet of an animal [13–17]. Black howler monkeys (*Alouatta pigra*) exhibit changes in their gut microbiota composition depending on which forest habitat type they inhabit [18]. Fruit availability within the habitat seems to be driving some of the most notable differences in gut compositional changes [18]. Specifically, a reduction in the abundance of Ruminococcaceae could be detected during periods of low fruit intake due to limited seasonal availability in primary and secondary forests [18]. Diet driven changes in the gut microbiota occur in wild house mice after being transferred into a laboratory environment and fed a captive diet. The composition of the microbiota changed from being rich in bacteria of the phylum Bacteroides to one dominated by phylum Firmicutes [19]. The observed change in dominant phyla present and relative abundance of specific bacterial groups is due to the increase of plant-derived nutrients found in the standard laboratory food and the lower amount of carbohydrates typically consumed in the wild [19,20].

Sex has been linked to differences in gut microbiota diversity in a few different mammalian species [7,21–23]. In humans, for example, a lower relative abundance of Bacteroidetes was found in women compared to men [7]. When looking at multiple strains of laboratory mice, some sex driven differences in the gut microbiota composition were unique to an individual strain suggesting that genetic variance also plays an important role in the variability of the microbiota in male and female mice [21].

Age of the host has not been identified to significantly impact the species composition of the microbiota once the individual has reached maturity [24]. Changes in the microbiota composition of young captive mice (9–15 weeks) occur through exposure to an increasing number of microbes from the environment, especially changes in diet, which would suggest that the surroundings play an important role in microbiota establishment and composition [25].

Nematode infections and other gut parasite burdens have all been shown to impact microbial diversity [13,26]. Infection with the gastrointestinal nematode *Heligmosomoides polygyrus* leads to an increase in the relative abundance of Lactobacillaceae [27]. Captive rats infected with rat tapeworm (*Hymenolepis diminuta*) had an increased relative abundance of *Clostridium* species, whilst *Bacillus* species had a reduced abundance compared to uninfected animals [26]. The mouse whipworm (*Trichuris muris*), frequently found in the caecum, is associated with a decrease in the phylum Bacteroidetes whilst increasing the abundance of Lactobacillaceae [6,28]. Another parasite frequently found in the caecum is the mouse pinworm (*Syphacia oblevata*) which has been associated with a decrease in *Lactobacillus* (phylum Firmicutes) [29].

Laboratory-based studies often consider the variables which may influence microbiota composition independently from one another; however, using a wild rodent population allows the exploration of how different effector variables combine to influence the composition of the gut microbiota

The sampling site of the present study, the Isle of May, lies approximately 8km off the south-east coast of Scotland in the Firth of Forth. The island is approximately 1.6km long and 0.5km wide [30,31]. The only terrestrial mammal species known to be living on the island are rabbits (*Oryctolagus cuniculus*) and a population of house mice [30]. The mice live in a range of different habitats throughout the island from shrubs and low grasses, to cracks in rocks and stone walls, to the boulders at the shallow northern end of the island and can even be found inside of puffin (*Fratercula arctica*) and rabbit burrows [30]. The home ranges of the mice have been studied and found to be largely overlapping, forming an island-wide panmictic unit with random mating occurring [31]. Mice have preferred, but not exclusive, ranges to one another and those living in open or unprotected habitat exhibit a greater tendency to roam compared to individuals living in more covered areas [31]. Males and females are not known to differ in their home ranges and movement patterns [31].

Earliest records of mice populations on the Isle of May date back to the late 19^th^ century [30]. In the 1980s seventy-seven mice from the island of Eday (Orkneys) were introduced to the existing population on the Isle of May [32]. Six months after the introduction, alleles from the Eday mice had introgressed into the resident population and were found in individuals from all trapping locations on the island, suggesting that successful hybridisation and free gene flow had occurred across the entire island [32]. This is supported by observations from re-trapping studies showing that mice move across wide ranging territories which overlap across the island [31] and suggests that the mouse population on the Isle of May is able to freely interbreed [32].

The mice on the Isle of May offer a unique opportunity to study factors influencing the composition of the gut microbiota in the same host species as commonly studied in the laboratory. These mice live wild and not in association with humans in comparison to those on the mainland which largely live a commensal existence [33].

We aimed to investigate the association of environmental and host variables with gut microbiota composition at the operational taxonomic unit (OTU) level, and found only trapping location and sex of the host to be significantly associated with the OTU composition of the gut microbiota. The environmental variables dominant vegetation, geographical distances among traps and trapping site locations, and the host variables age, sex, condition and infection status were tested and showed no significant associations with the microbiota composition.

## Materials and methods

The following methods were approved by the University of Nottingham Animal Welfare and Ethical Review Body and comply with the UK’s Animals (Scientific Procedures) Act of 1986.

Wild *M*. *m*. *domesticus* were caught on the Isle of May, Firth of Forth, Scotland, in October 2015. A total of 11 sampling sites were used as described by Taylor et al. 2019 [34]. At each site 15 to 20 traps were laid using 10 separate transect points, each with one or two traps depending on overall size of trapping site. Transect points were between 1 and 3 meters apart. We predominantly used Longworth (Longworth Scientific Instrument Co., Oxford, UK) or Ugglan (Granhab, Gnosjö, Sweden) small animal traps and a small number of home-made “Jordan” traps [35] constructed from drain pipes with a trapping mechanism inspired by Ugglan traps. Traps were filled with hay and baited with a handful of birdseed and then checked twice a day, morning and late afternoon, across a total of 4 days.

Each sampling site was categorised by plant species according to its dominant vegetation, based on the most recent available survey of the island (van der Wal, unpublished). Dominant plant species were Yorkshire fog *Holcus lanatus* and sea campion *Silene maritima*, or the category “Both” was used for sites with equal abundance of these two plant species.

All captured mice were sexed and weighed; females displaying signs of pregnancy or lactation were released. A subset of animals per site were randomly selected to be culled to ensure an even distribution of samples across the different sampling locations. Individuals selected for culling were euthanized using rising levels of CO_2_ and death confirmed by exsanguination, in accordance with Schedule 1 of the Animals (Scientific Procedures) Act 1986. Immediately following euthanasia, mice were weighed and then dissected. The gastrointestinal tract was removed and stored in 80% ethanol before detection of endoparasites under a dissection microscope. Eyes were removed and stored in 80% formalin; lenses were later removed, dried and weighed to estimate age in days as described in Rowe et al. 1985 [36]. Mass of the eye lenses has been previously used to estimate age as the lenses grow throughout the animal’s lifespan [37]. Eye lens mass as predictor of age was compared to body mass, body length, tail length, foot length and ear length in corn mice (*Calomys musculinus*) and was found to be the most reliable measure of age compared to the other variables [38]. The use of eye lens weight converted into age in days successfully allowed infants, juveniles and three age-classes of adults to be distinguished in wild house mice (*M*. *m*. *domesticus*) [36,39].

A single sample of caecum tissue and content was collected from each individual, such that variation among these caecum samples (hereafter, just “samples”) is representative of variation among individuals. The sample was immediately snap frozen to preserve DNA yield and stored at -80°C. DNA was extracted from samples using the PowerSoil DNA Isolation Kit (MO BIO Laboratories, Inc.). This included a 20 minute bead beating step utilising the supplied PowerBead tubes. Out of the 100 samples, 80 samples were selected after a two-step PCR amplification was performed for quality control. The first round PCR utilised the bacterial 16S rRNA primers 515F and 806R (16S region V4 primers [40]). Cycling conditions were 95°C for 3 minutes, followed by 25 cycles of 98°C (20 seconds), 60°C (15 seconds), 72°C (20 seconds), and a final extension at 72°C for 5 minutes. The second stage PCR used Illumina Nextera PCR library preparation kit containing Illumina adapters. The cycling conditions were 95°C (3 minutes), followed for 15 cycles at 95°C (30 seconds), 55°C (30 seconds), 72°C (30 seconds) followed by extension at 72°C for 5 minutes. Following the two step PCR, MiSeq sequencing was carried out using MiSeq V3 reagent kit and 500 sequencing cycles. Samples with less than 15,000 sequences were removed to minimise the loss of sequence information associated with sub-sampling the dataset. Sequences were clustered at 97% identity threshold and chimera removal was performed using USEARCH/UCHIME [41]. The resulting OTU sequences were classified in Mothur [42] using the Silva reference files [43], with a confidence threshold of 80%, and eukaryotic, archaeal, mitochondrial and plastid sequences, along with those which were unclassified at the domain level, were removed. Paired-end reads were quality checked using FastQC [44], low-quality regions trimmed and only sequences with Q30 >90% were kept, and finally, sequences were merged using BBmerge [45].

All data analysis was carried out using R (R version 3.5.0; 2018-04-23) using the packages Phyloseq [46] and Vegan [47]. Differences in number of reads among caecum samples was accounted for by dividing each sequence count by the total number of reads in that sample, yielding relative abundance measures [46], except in the case of differential expression analysis for which raw sequence counts were used [48].

Body weight residuals were calculated from a linear model using the raw body weight as the response variable and snout to vent length (including second and third order polynomial terms) and sex as predictor variables. From this, negative or positive residual values identified animals which were lower or higher than average weight (respectively) given their length and sex. The residual values were used as a measure of condition as we made the assumption that heavier than normal animals will have better nutritional reserves than abnormally light individuals. Weight residuals have previously been used by others as a method of assessing overall condition of the animal [49].

Alpha diversity, the number and evenness of different OTUs within a sample, was calculated using both Shannon and Simpson indices. Results obtained from both the Shannon and Simpson indices were qualitatively similar; we only present the Shannon index outputs here. A linear model was used to investigate the associations between Shannon index and host variables: sex, age (based on eye lens weight and converted into age in days), body weight residuals (as a proxy of condition), the trapping location of the host, *T*. *muris* intensity (log transformed), *S*. *obvelata* intensity (log transformed) as well as vegetation at trapping site. Furthermore, the potential associations between *S*. *obvelata* intensity, on an individual level, and the phylum Firmicutes was analysed using a linear model, which included the logged number of OTUs belonging to the phylum Firmicutes as dependent variable and *S*. *obvelata* counts as the independent variable, and similarly the potential relationship between the phylum Bacteroidetes and *T*. *muris* was investigated.

Beta diversity, the similarity of OTU composition among caecum samples, was measured using UniFrac distance, a measure which takes phylogenetic distances into account [50], and Bray-Curtis dissimilarity index. The Bray-Curtis index and UniFrac distance measures agreed with each other with only one exception; in all other cases only the result from Bray-Curtis is shown for simplicity. The one instance of difference in outcome between the two measures is highlighted in the results section. Beta diversity was compared to host and environmental variables using permutational multivariate analysis of variance [51] based on the distance tables. This was done using the Adonis function in R [47] with sex, age, *T*. *muris* and *S*. *obvelata* infection (log transformed), trapping location and health condition of the animals–measured using weight residual as a representative of condition–as predictor variables and Bray-Curtis dissimilarity index or UniFrac distance index as the response. P values were estimated by comparing pseudo-F values against 999 random permutations of the data [51]. We then followed up the significant results from our analysis of beta diversity by testing for differential expression among OTUs in the relevant variables (trapping location and sex) using the DESeq2 package in R [52]. To identify OTUs driving the difference between males and females a Wald test was performed on the log fold change data. To analyse the differences in OTUs among trapping sites a likelihood ratio test was performed comparing a full model which included both sex and location and a reduced model which only contained sex. An analysis of deviance captures the difference in likelihood between the full and the reduced model.

To investigate the effect of physical distance between traps, a Mantel test was performed comparing distance matrices from the GPS trap location data and the microbiota community data from the caecum samples.

Principal Coordinates Analysis (PCoA) was used as a distance based ordination method to produce PC1 and PC2 using the Bray-Curtis distance to use for plotting the variables.

## Results

Following removal of unusable samples, caecal microbiota data were obtained from a total of 79 mice.

**Fig 1:**
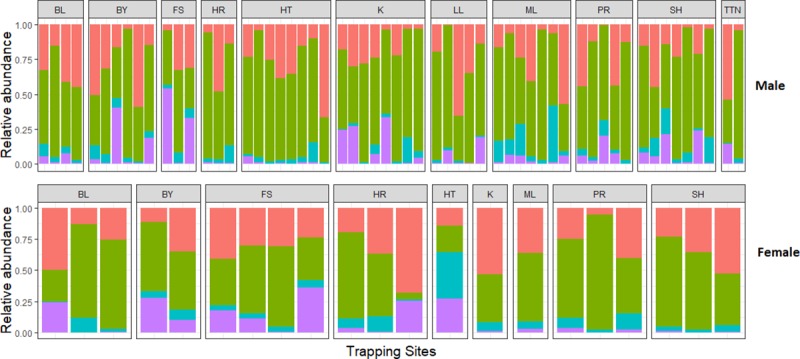
The relative abundance of bacterial phyla in the microbiota of wild mice. The different trapping sites are along the x-axis and relative abundance of most common phyla along the y-axis. The figure is divided into two panels containing females in the top panel and males in the bottom panel. The phylum Bacteroidetes is represented in red at the top, Firmicutes are shown as green, Proteobacteria at the very bottom are shown in purple. Any other phyla were grouped together and are represented in blue.

Sequences obtained were clustered into a total of 3006 OTUs with 579 OTUs having 100 or more reads across all samples, and the highest number of reads being 235268. The mean number of reads per caecum sample/mouse was 68839. Across all samples the phyla Firmicutes, Bacteroidetes and Proteobacteria were the three most abundant at an average of 53%, 30.3% and 9.9% respectively (Fig 1). Alpha diversity did not vary significantly with sex, age, trapping location, weight residuals of the host, *T*. *muris* and *S*. *obvelata* presence or vegetation at trapping site (Table 1). This suggests a stable alpha diversity across our population (Linear regression: residual standard error (SE) = 0.57, degrees of freedom (df) = 63, F = 0.53, p > 0.05).

**Table 1:** Variables of the individual host as well as its environment compared to microbiota alpha diversity using a linear model. None of the variables tested showed a significant association with alpha diversity index (Shannon index).

Variable	Standard Error	p-value	Parameter estimates
Trapping Location	0.89	0.63	-0.41
Sex (Male)	0.061	0.93	-0.37
Age	0.046	0.91	-0.027
Weight Residuals	0.016	0.69	-0.41
*Trichuris muris* intensity (log)	0.12	0.21	-0.14
*Syphacia obvelata* intensity (log)	0.032	0.43	0.78
Dominant Vegetation (*Silene maritima*)	0.45	0.89	0.12

### Trapping location has a significant association with OTU composition

**Fig 2:**
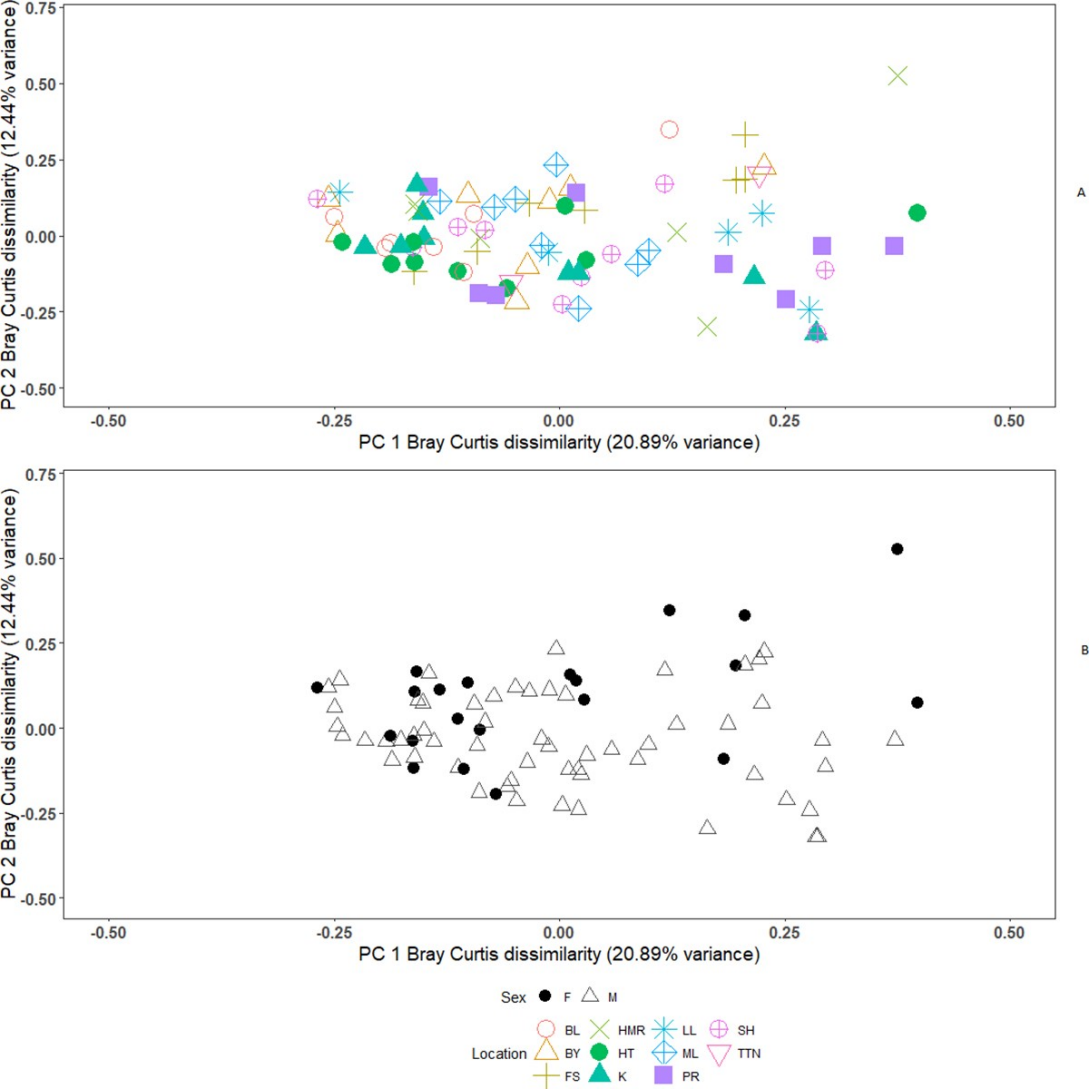
**A. Bray-Curtis dissimilarity index across trapping locations.** Bray-Curtis dissimilarity index was used to calculate the distance between individuals’ OTU composition and plotted using PC1 and PC2 from PCoA. PC1 represents 20.89% of the variance while PC2 represents 12.44% of the variance. Locations are shown as separate colours and shapes to show differences within and between sites. Trapping sites were represented by their respective abbreviations (BL = Burnet’s Leap, LL = Low Light, TTN = Three Tarns Nick, HR = Holyman’s Road, ML = Main Light, HT = High Tarn, FS = Fluke Street, BY = Byres, K = Kettle, PR = Priory, SH = South Horn) and are the same as used in Taylor et al. [34]. The OTU composition varies significantly among trapping locations across the island. **B. Bray-Curtis dissimilarity index between male and female mice.** The same PCoA was used to display the difference between male and female mice. Females are shown as black circles, males as grey triangles. A significant association between sex and microbiota beta diversity was found (Adonis: R^2^ = 0.02, p = 0.04.

OTU composition showed a significant association with trapping location when using a permutational multivariate analysis model based on Bray-Curtis dissimilarity index. The model was used to test associations between the OTU composition of the host, representative of beta diversity, and the predictor variables sex, age, trapping location, *T*. *muris* and *S*. *obvelata* infection (log transformed) and overall weight residuals (as a measure of condition) (Table 2). Trapping location accounts for 15% of total microbiota OTU variation (R^2^ = 0.15, p = 0.007, df = 10; see Fig 2). Trapping location is the environmental variable that explains the most variance in OTU composition. A further 2% of overall variation can be explained by sex (Table 2).

**Table 2:** Environmental and host variables were compared to OTU composition (Bray-Curtis index) using a permutational multivariate analysis (Adonis).

Variable	R^2^	p-value	Degrees of freedom
Trapping Location	0.15	0.009 *	10
Sex	0.018	0.034 *	1
Age (in days)	0.01	0.42	1
Weight Residuals	0.01	0.89	1
*Trichuris muris* infection (log)	0.013	0.51	1
*Syphacia obvelata* infection (log)	0.012	0.58	1

* significant at p < 0.05

We used differential expression analysis (DESeq2) to investigate which taxa were driving the observed differences in microbiota among trapping locations. Trapping location was significantly associated with relative abundance of 123 OTUs. A large proportion of these OTUs (39%) belonged to the Lachnospiraceae, including the genera *Roseburia*, *Dorea*, *Clostridium-XlVa and Clostridium-XlVb*. Overall, Lachnospiraceae comprised only 9.9% of the OTUs recorded in this study, indicating that they were strongly over-represented in the associations with trapping location (Table 3).

**Table 3:** The bacterial families identified using a likelihood ratio test driving the difference between trapping sites and the number of OTUs belonging to each family.

Family	No. of OTUs	Proportions in overall data (%)
Bacteroidaceae	3	0.6
Clostridiales	11	3
Firmicutes (unclassified)	6	1.3
Helicobacteraceae	2	0.7
Lachnospiraceae	48	9.9
Porphyromonadaceae	5	1.1
Ruminococcaceae	10	3.4
Rikenellaceae	3	0.4
Others	7	5.9
Unclassified	29	73.3

We did not find any significant association between GPS trap location data and the microbiota composition of the caecum (Mantel statistic r = 0.03, p = 0.27).

### OTU composition of individuals shows potential differences between the sexes but no differences driven by age

Using the Bray-Curtis dissimilarity index a significant difference in OTU composition was found between male and female mice (Adonis, R^2^ = 0.02, p = 0.04, df = 1; see Fig 2B). However, using weighted UniFrac distance instead of the Bray-Curtis index no significant association was found in the model using the same variables (Adonis, R^2^ = 0.014, p = 0.17, df = 1). Age, parasite burden and weight residuals all showed no significant association with the beta diversity (Table 2).

Differential expression analysis was utilised to establish which OTUs occurred at significantly different proportions in females compared to males (Table 4). Out of 14 OTUs which were found to be significantly more abundant in males compared to females, 7 belonged to the family Lachnospiraceae, specifically belonging to the genera *Robinsoniella*, *Blautia*, *Clostridium-XlVb* and *Clostridium-XlVa*. Two of the OTUs belonged to the Ruminococcaceae family, one of which was identified as part of the genus *Pseudoflavonifractor*.

**Table 4:** DESeq2 analysis was performed to identify OTUs at Family level which had higher relative abundance in males compared to females and vice versa.

More abundant in males		More abundant in females	
Family	No. OTUs	Family	No. OTUs
Lachnospiraceae	7	Bacteroidaceae	4
Ruminococcaceae	2	Clostridiaceae	4
Erysipelotrichaceae	1	Lachnospiraceae	5
Clostridiales	1	Porphyromonadaceae	4
Peptostreptococcaceae	1	Proteobacteria	1
Rikenellaceae	1	Unclassified	7
Unclassified	1	Other	4

Females had a total of 31 significantly more abundant OTUs compared to males, 5 of which belonged to the family Lachnospiraceae. Four of the OTUs which were more abundant in females belonged to the family Clostridiaceae and another 4 OTUs belonged to the Porphyromonadaceae.

### Infection with parasites does not significantly associate with microbiota composition

The endoparasite species *T*. *muris* and *S*. *obvelata* occurred in a majority of mice (77.2% infected with one or both parasites). The endoparasite *Aspicularis tetrapetra* and *Hymenolepis nana* were each only detected in a single individual and were not included in any further analyses. The only ectoparasites found were the fur mites *Radfordia affinis*, *Myocoptes musculinus* and *Myobia musculi* and the northern rat flea (*Nosopsyllus fasciatus*) (Table 5).

**Table 5:** The percentage of infected mice (prevalence) for the different, commonly found parasites.

Parasite species	Prevalence
*Trichuris muris*	40.5
*Syphacia obvelata*	57
*Radfordia affinis/ Myocoptes musculinus/ Myobia musculi*	97.5
*Nosopsyllus fasciatus*	22.8

Using a linear model we specifically tested for changes in the abundance of OTUs belonging to Firmicutes against observed *S*. *obvelata* presence, sex and age of the host but found no significant associations (residual SE = 0.124, df = 73, F = 1.38, p > 0.05). We similarly tested for an association between Bacteroidetes abundance and *T*. *muris* presence but found no significant associations among variables (residual SE = 0.13, df = 73, F = 1.38, p > 0.05).

## Discussion

Here, we report a significant association between the beta diversity of the wild mouse gut microbiota and the trapping location of the individuals. Dominant vegetation species at trapping locations did not directly impact the composition of the gut microbiota; however, the exact vegetation composition may differ among our trapping locations and could potentially lead to differences in dietary profiles of individual mice.

Differences in the microbiota composition of the gut in wild mice have commonly been shown to be associated with geographical location, however, sampling distances in wild studies range from up to 100km between trapping sites [13] to different countries [14]. This means results will be confounded by local differences, such as host genetics, local infections and vegetation, among mouse populations [13,14,53]. The furthest distance between our trapping sites on the Isle of May was 1.5km, on average our sites were spaced 100m apart and the mouse population is believed to be able to roam freely across the whole island with male and female territories spanning between 100m^2^ up to 400m^2^ [32]. Despite the small spatial scale of the present study, trapping location was significantly associated with gut microbiota composition in our mouse population. There was, however, no significant association between the geographical distance among the individual traps and the microbiota composition. Therefore, while we observe geographical variation in gut microbiota, this variation is not directly related to distance.

There are several potential explanations to account for the small scale locational differences and the observed variation in caecal microbiota composition. Animals living in close proximity are likely to be both genetically related and exposed to similar bacteria in the environment. Both male and female mice tend to remain within their home ranges with close overlap in ranges between small groups or pairs of mice [31].

Vertical transmission can occur from mothers and close litter mates [8]. Similarly, individuals which are directly related to one another may share a greater proportion of gut microbiota composition than unrelated animals [54].

If we had detected a significant association between inter-trap distances and microbiota composition, this would have provided support for the hypothesis that genetic diversity and familial relationships explain the differences in microbiota among sites. On the other hand, we still cannot rule out a possible role for genetic diversity here, as local patterns of genetic diversity do not necessarily correlate perfectly with distance. For example, Suzuki et al. found that variation in microbiota composition across several wild mouse populations was more strongly associated with genetic variation than with geographic distance [55]. Alternatively, there could be environmental differences among sites that are independent of our measure of dominant vegetation type, such as availability of other food sources, which might explain the inter-site differences. More detailed genetic and environmental data is required to distinguish between these two hypotheses.

OTUs belonging to the families Lachnospiraceae and Ruminococcaceae were identified as significantly shaping differences in microbiome composition among trapping sites. Both Ruminococcaceae and Lachnospiraceae are involved in the breakdown of complex plant materials [56], such as cellulose, and are able to process plant polysaccharides [57] and have been associated with changes in diet in response to high plant and fibre content [18,52,53]. The specialised role of these bacterial families could suggest a significant difference in available fibre and vegetation among the trapping sites of the present study which drives the difference in microbiota communities observed. Another study on wild house mice identified *Bacteroides* (Bacteroidaceae), *Robinsoniella* (Lachnospiraceae) and *Helicobacter* (Helicobacteraceae) as being significantly impacted by geographical variation of trapping site [14], all of which belong to families also related to trapping location in this study.

The composition of the gut microbiota in wild mice captured on the Isle of May is made up of the same common phyla as other rodent studies: Bacteroidetes, Firmicutes and Proteobacteria predominated and are the most common bacterial phyla across most mammalian species [13,14,19]. Firmicutes have been found to be the most common phylum within wild rodent microbiota compared to laboratory-reared individuals which often display higher levels of Bacteroidetes [13,58]. This difference in dominant phylum has been shown to be associated with differences in available diet or even access to food or periods of fasting which have been shown to potentially increase the relative abundance of Bacteroidetes [59]. The diet of many wild rodents changes with season, from plant and seed based to insect based depending on food availability [53]. In particular *Lactobacillus* was found to be significantly increased in a population of wild wood mice during spring compared to autumn and winter, which corresponds to an increase in available insects, whilst *Alistipes* and *Helicobacter* were shown to be more abundant in autumn than in spring, which coincides with an increase in available seeds and a possible shift in the mouse diet [53]. It was found that the amount of food consumed differed between spring and autumn which may also impact on the composition of the gut microbiota [53]. Our mice were all trapped in October of 2015 so the differences we observed cannot be due to seasonal variation.

The age of the host did not show any significant association with microbiota composition, which is unsurprising as both human and murine studies have suggested stability of the microbial composition over time within these host species [54,60]. Significant differences in microbiota compositions are only noted during infancy in the first few weeks and months of life [61], however, as we only sampled individuals post-weaning it is unlikely that we would have sampled individuals young enough to note a difference with age.

We hypothesised that sex would show associations with differences in microbiota composition but found that it had no significant impact on the alpha diversity of the gut microbiota. In the present study sex of the host had a possible association with the gut microbiota when using the Bray-Curtis index, but this association was not significant when using UniFrac distance. Sex driven differences in the composition of the gut microbiota have been found in mice but are often reported as weak [53] to non-existent or negligible [13,29]. The difference in OTU composition between male and female mice might be explained by differences in their diet. Wild pikas (*Ochotona curzoniae*) were found to show distinctly individualised foraging preferences even within the same sampling location and had their own individual dietary profile which in turn impacted their gut microbiota composition [62]. Though foraging differences between the sexes were not specifically investigated it is possible that food profiles between male and female wild rodents do differ and cause a change in gut microbiota composition [22,62]. On the other hand, there is no specific evidence that male and female mice exhibit different foraging behaviour.

There are differences in the effect diet has on the microbiota between sexes in both fish and mice [22]. Males showed an overall greater change in OTU abundance after a change in their diet than females [22]. A weak relationship between diet driven changes of the microbiota and host sex can also be detected in mammals such as mice [22]. These findings are based on the assumption that males and females consume the same diet.

Another possible explanation for compositional differences between the sexes would be hormone driven changes leading to differences in the microbiota [21,63]. Hormonal effects are difficult to establish as they were shown to be obscured by variation in host genetics and could only clearly be established in individuals belonging to the same genetic strain [21]. The present study cannot conclusively establish a significant association between beta diversity and sex as this association was not observed when utilising weighted UniFrac distance. Whilst Bray-Curtis dissimilarity compares the differences in relative abundance between two sites, or two individuals, weighted UniFrac also considers phylogenetic distances. The lack of significant association with UniFrac distances may indicate that while OTU counts differ between males and females, the taxa present are similar and therefore phylogenetic distances are not large.

No association was found between parasite infections and the gut microbiota composition in the present study. Based on the results from other studies [29,53,64] we expected to see that individuals infected with parasites–in particular *S*. *obvelata* and *T*. *muris*–would show differences in their microbiota composition compared to non-infected individuals. A study on wild mice (*Apodemus flavicollis*) showed an alteration in the gut microbiota composition to be associated with *Heligmosomoides polygyrus*, *Syphacia spp*. and *Hymenolepis spp*. infections [29]. The presence of *T*. *muris* infection in mice has also been associated with a relative increase in the abundance of the *Lactobacillus* genus in the gut [6]. Increased abundance of bacteria in the large intestine has been positively associated with the hatching of *T*. *muris* eggs suggesting that this gastrointestinal parasite requires the physical structure provided by a number of different bacteria [64].

We did not find an association between *T*. *muris* and *S*. *obvelata* infection and the alpha or beta composition of the microbiota in Isle of May mice. This may be due to sampling only one site of the gastro-intestinal tract. The presence of nematodes in the gut has been shown to influence the gut microbiota in a very gut site specific manner [29]. One of the most notable interactions between parasite and microbiota composition occurred in the small intestine of individuals infected with the tapeworm *Hymenolepis nana* where there was an increase of the Bacteroidetes species S24-7 [29]. Similarly, laboratory reared C57BL/6 mice showed significant shifts in their microbiota composition when artificially infected by *Heligmosomoides polygyrus bakeri* [27]. The changes in the microbiota composition could, however, only be identified in the ileum which is the helminth’s typical niche in its host [27]. Microbiota samples from the caecum of the infected host did not show any compositional changes suggesting that helminth infection affects the immediate surrounding gut microbiota but not necessarily throughout the gastrointestinal tract [27,29]. By sampling multiple sites within the mouse gut we may in the future be able to establish associations between different gut endoparasite infections and the gut microbiota composition.

Collecting detailed vegetation data at our sites as well as analysing gut contents to identify the diet of mice would help in establishing causality between dietary differences and microbiota compositional changes. Additionally, collecting longitudinal data would give a greater insight into variation over time allowing observation of changes occurring in host’s biology such as in parasite infections or body condition and comparing them to differences in microbiota composition. As well as determining causality for geographical differences in the microbiota, longitudinal monitoring could offer an insight into fitness benefits of microbiota composition when considering reproductive success and longevity of the host.

In the present study the relative abundance of bacterial OTUs was utilised to investigate associations with environmental and biological variables. In some contexts, analysing the absolute abundance of bacterial taxa can give greater ability to distinguish among individuals. For example, differences between patients suffering from Crohn’s disease and healthy individuals were associated with absolute changes in bacterial numbers [65]. However it was not possible to examine differences in absolute abundance of bacteria with the data gathered in this study as cell counts and qPCR assessment of bacterial loads would have been needed for this type of analysis. Furthermore, by focusing on relative abundance we have been able to compare our results more directly to similar studies as it is currently a more common approach.

In summary, we found that there is significant variation in gut microbiota diversity even over very small distances which highlights geographical location and relative distance between sampling sites as an important consideration for future microbiota studies.

## Supporting information

S1 Data Dissection data Isle of May mice. A spreadsheet containing data associated with the dissection of the mice captured on the Isle of May.
(CSV)

S2 Data OTU counts table. A spreadsheet containing count data for each OTU across all individual samples.
(CSV)

S3 Data OTU taxonomy table. A spreadsheet listing taxonomic classification for each identified OTU.
(CSV)
